# Primary Ewing’s Sarcoma of the temporal bone in an infant

**Published:** 2015-04-01

**Authors:** Kourosh Goudarzipour, Shahin Shamsian, Samin Alavi, Kazem Nourbakhsh, Roxana Aghakhani, Zahra Eydian, Mohammad Taghi Arzanian

**Affiliations:** 1 Pediatric Congenital Hematologic Disorders Research Center, Shahid Beheshti Medical University, Tehran, Iran; 2 Department of Pathology, Shahid Beheshti Medical University, Tehran, Iran

**Keywords:** Ewing’s sarcoma skull, Temporal bone, Computed tomography, Chemotherapy

## Abstract

**Introduction** **: **Ewing’s sarcoma is the second most common primary malignant tumor of bone found in children after Osteosarcoma. It accounts for 4–9% of primary malignant bone tumors and it affects bones of the skull or face in only 1–4% of cases. Hence it rarely affects the head and neck.

**Subject and Method** **: **In this case report, we describe a case of primary Ewing's sarcoma occurring in the temporal bone. The tumor was surgically excised, and the patient underwent chemotherapy for ten months.

**Results** **: **Neither recurrence nor distant metastasis was noted in these 10 months after surgery but about 18 months after surgery our patient was expired.

**Conclusion** **: **Although the prognosis of Ewing's sarcoma is generally poor because of early metastasis to the lungs and to other bones, a review of the article suggested that Ewing’s sarcoma occurring in the skull can often be successfully managed by intensive therapy with radical excision and chemotherapy. This result was supported by the case reported here.

## Introduction

 Ewing's sarcoma is a primary bone cancer that affects mainly children and adolescents. It's one of a group of cancers known collectively as the Ewing sarcoma family of tumors. It's the second most common bone cancer in children, but it's also relatively uncommon. It accounts for only 1% of all childhood cancers. Although it can occur at any age, it rarely occurs in adults over the age of thirty. Ewing's sarcoma represents 4% to 9% of primary malignant bone tumors^1^^,^^2^ and is most frequently seen in the long bones or pelvis. Primary Ewing's sarcomas occurring in the skull are exceptionally rare.^3^ The prognosis of Ewing's sarcoma is often poor because of early metastasis to the lungs or to other bones.^4^ However, a review of articles suggested that metastases are much less frequent and long term survival can be expected in Ewing’s sarcoma occurring in skull. 

## CASE REPORT

 Parents of a 9-month-old boy noticed a swelling in their infant’s right temporal region in November 2007. After three months he was referred to our hospital for indolent mass of skull, in initial physical examination cranial nerves were intact and there was no sign of lymphadenopathy and hepatosplenomegaly.

**Figure 1:**
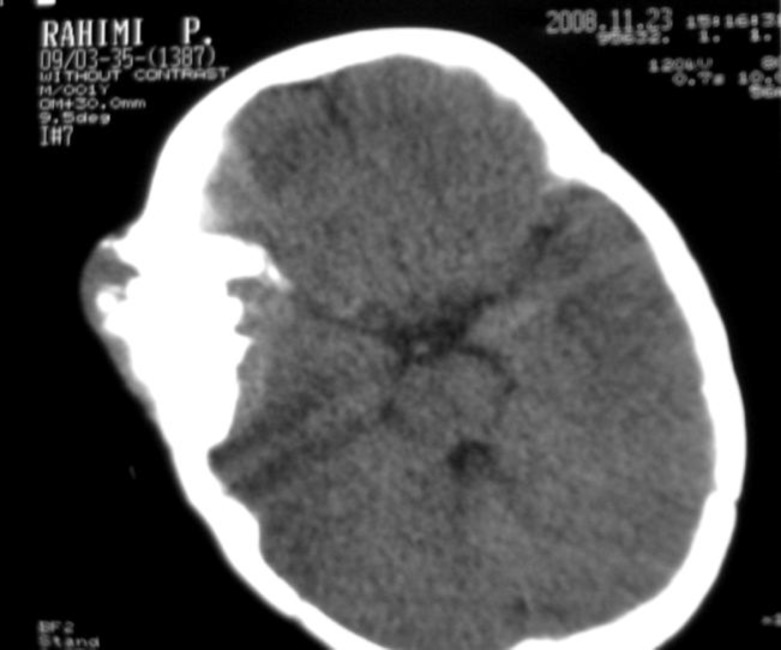
CT scans revealed an intracranial, well-defined, isodense mass, surrounded by a hypodense area and hyperostosis in the right temporal region adjacent to the tumor. Part of the tumor grew extracranially. The tumor was homogeneously enhanced by intravenous infusion of contrast medium, also brain Parenchyma was intact

 A three centimeters mass was palpated beneath the surface of the skin in the right temporal region. The mass was solid, immobile, and non-tender. The skull films were not available but skull graphies showed a well-defined bony mass in right fronto-parietal region and recommended performing bone biopsy. Computed tomography (CT) scans revealed an intracranial, well-circumscribed, isodense mass, surrounded by a hypodense area and hyperostosis in the right temporal region adjacent to the tumor. Part of the tumor grew extra-cranially. The tumor was homogeneously enhanced by intravenous infusion of contrast medium; also brain parenchyma was intact (Figure 1).

 The results of initial paraclinical studies are listed below: WBC: 4400/dl (PMN: 30%, Lymph: 66%, Mono: 3%), Hb: 10.4 mg/dl, Plt: 235000/dl, ESR: 18 mm/h. Biochemistry tests were normal.

Tumors that show good differentiation are generally easy to diagnose, but when a tumor is poorly differentiated, identification, morphological features is more difficult. Bone needle biopsy was performed but results showed normal specimen, according to the X-ray report, normal needle bone biopsy and to acquire samples for IHC and cytogenetic, the patient underwent surgical removal of the tumor on February 2008. A brown-colored, solid mass was totally removed, along with hyperostotic temporal bone and overlying temporal muscle. The cortical surface was readily separated from the tumor in all parts.

**Figure 2:**
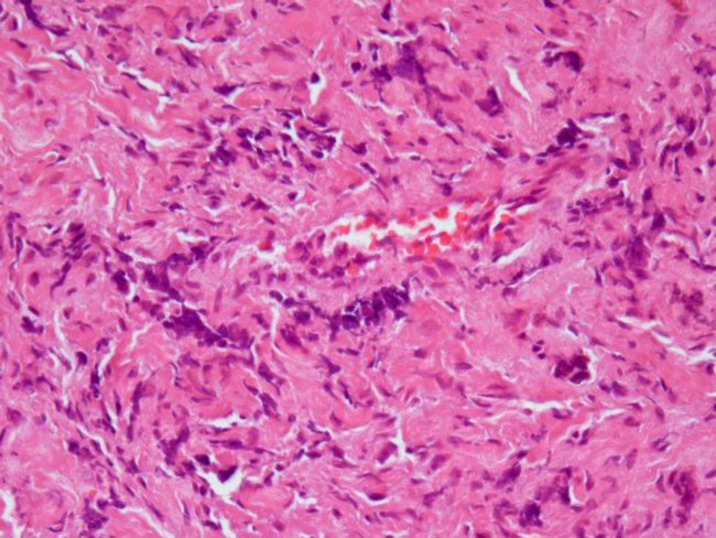
The tumor was composed of solidly packed lobular round cell pattern of striking uniformity. The individual cells possessed a rounded or ovoid vesicular nucleus. The cytoplasm was ill-defined, scanty and pale staining

 The tumor was composed of solid packed lobular round cell pattern of striking uniformity. The individual cells possessed a rounded or ovoid vesicular nucleus. The cytoplasm was ill-defined, scanty and pale staining (Figure 2).

Immunostaining for NSE (Neuron Specific Enolase) and CD99 (Mic-2) were positive. RNA was analyzed for t (11; 22) by reverse transcription followed by RT-PCR method, result was negative for t (11; 22) (q24; q12), EWS-FLI1 fusion transcript. 

 These findings were compatible with a diagnosis of Ewing's sarcoma. Other evaluations including chest, abdomen and pelvis computed tomography scan were normal. Whole body bone scan revealed increase uptake in temporal bone. After surgery, chemotherapy was begun by port cat system. The postoperative course was uneventful. 

Chemotherapy included Vincristine 2 mg/m², Doxorubicin 75 mg/m², Cyclophosphamide 1200 mg/m² followed by MESNA alternating with Ifosfamide 1800 mg/m²/day for five days and Etoposide 100 mg/m²/day for five days (We adjusted 25% doses of drugs till he became one year old). The courses are administered every three weeks for total 17 courses with planned treatment duration of 49 weeks. Doxorubicin is substituted by Actinomycin D 1.25 mg/m²/dose when total Doxorubicin dose is reached to 375 mg/m² (after 5 cycles). Radiotherapy was not done because patient was less than 2 years old. Ten months after surgery, the evidence for re-growth of tumor or distant metastasis was negative. After 18 months of the last therapy, our patient referred to hospital because of pneumonia infection. Due to its infection, antibiotic therapy was started for him but in spite of all efforts he was expired within a few days.

## Discussion

 The primary location of Ewing's sarcoma is generally the long bones or the trunk bones. These tumors tend to break through the cortex of the bone of origin into the adjacent soft tissues.

The majority of patients with Ewing's sarcoma complain of pain and swelling in the area of the tumor. Compromised function of the affected part, fever, tenderness, local heat, and a palpable mass are also encountered.^5^

The duration of symptoms before presentation ranges from two weeks to two years.^6^ The skull is a rare primary site for Ewing's sarcoma.^7^ Sixteen cases of primary Ewing's sarcoma of the cranium have been presented in the literature. ^6^^,^^7^^,^^8^^,^^9^

 Vaccani et al. reported 70 cases of Ewing’s sarcoma between 1986 - 1996, among them five cases suffered from Ewing's sarcoma of head and neck (7.1 %) and the age of presentation ranged from 7.5 to 14 years. An enlarging mass in the mandible is the most frequent presentation, in none of them temporal bone was involved. Three of five patients died of metastatic disease and two are alive with no evidence of disease.^10^

 Kuzeyli et al. reported a primary Ewing sarcoma of temporal bone in a case of ten years-old girl. She presented with palpable mass on the right fronto-temporal region and proptosis of the right eye but six months later she died because of lung metastases.^5^

 Ewing’s sarcoma is more common in second decade of life and temporal bone involvement is very rare. Ernest et al. reported one case of Ewing’s sarcoma in a seven months-old white girl in right leg. The mass was not mobile and appeared to arise definitely from the tibia, not from the overlying soft tissue. No metastatic lesions were noted in lungs fields. She deteriorated rapidly and expired six month after the first appearance of her symptoms.

Our case due to age of the patient and location of the tumor is a very unique one in literature.   

Although according to the young age treatment was so hard and radiotherapy was excluded due to its side effect and also chemotherapy has more dangerous side effects, in consequence Ewing’s sarcoma has poor prognosis in less than one year-old children. Our case has no evidence of recurrence and metastasis after ten months and the therapy was suitable for him but unfortunately he was expired after 18 months because of pneumonia that was resistant to antibacterial therapies.
